# “I’ve got a spring in my step” participants experience of action observation therapy and eccentric exercises, a telehealth study for mid-portion Achilles Tendinopathy: a qualitative study

**DOI:** 10.1186/s13047-023-00619-x

**Published:** 2023-04-11

**Authors:** Deirdre Ryan, Ebonie Rio, Grainne O’Donoghue, Cliona O’Sullivan

**Affiliations:** 1 UCD School of Public Health, Physiotherapy and Sports Science, Dublin, Ireland; 2 School of Allied Health, La Trobe University Melbourne, Melbourne, Australia

**Keywords:** Mid-portion achilles tendinopathy, Action observation therapy, Qualitative research, Telehealth

## Abstract

**Background:**

Quantitative research has dominated the field of Achilles Tendinopathy. The use of qualitative research allows in-depth exploration of participants’ perspectives, offering great insight in the evaluation of a trial’s processes, particularly when exploring a novel intervention such as Action Observation Therapy combined with eccentric exercises which has not been previously researched. This study aimed to qualitatively explore participants’ experiences of partaking in a telehealth study including the acceptability of the intervention, motivators for participation, and perspectives on the trial processes.

**Method:**

A thematic analysis as guided by Braun and Clarke was used to analyse the semi-structured interviews conducted on a purposive sample of participants with mid-portion Achilles Tendinopathy who recently completed a pilot feasibility study. The study adhered to the criteria for reporting qualitative research guidelines (COREQ).

**Results/Discussion:**

Sixteen participants were interviewed. The five themes identified were: (i) *The impact of Achilles Tendinopathy is commonly not prioritised* with ‘*The acceptance and minimisation of pain’* as a sub-theme (ii) *Therapeutic alliance has the greatest impact on support* (iii) *Factors which* influenced *adherence* (iv) *Action Observation Therapy is valued and recommended* (v) *Recommendations for future interventions.*

**Conclusion:**

This study provides insightful recommendations around; exploring the use of Action Observation Therapy in Achilles Tendinopathy, the relative importance of therapeutic alliance rather than mode of therapy delivery, and that sufferers of Achilles Tendinopathy may not prioritise health seeking for this condition.

**Supplementary Information:**

The online version contains supplementary material available at 10.1186/s13047-023-00619-x.

## Introduction

Despite the growth in research, Achilles Tendinopathy is recognised as a difficult condition to successfully rehabilitate, with chronic symptoms persisting in 60% of individuals treated conservatively [1]. The use of qualitative research in feasibility studies for randomised controlled trials has become increasingly common [2], however a predominance of quantitative research exists within the field of Achilles Tendinopathy, with only four studies employing qualitative methodologies to explore trial interventions or the experience of living with this condition [3–6].

A recent pilot feasibility study was conducted exploring the effect of Action Observation Therapy combined with eccentric exercises in persons with Achilles Tendinopathy. This is the first time Action Observation Therapy has been explored in this population. Action Observation Therapy is based upon the discovery of the mirror neuron system in the brain, these neurons activate when one observes an action in a similar pattern to as though the individual is executing the action themselves [7]. The activation of these neurons has been shown to positively contribute to rehabilitation programmes by facilitating motor learning, enhancing specific motor skills and decreasing the severity of pain in neurological and musculoskeletal populations [8–10]. Typically Action Observation Therapy involves watching a video of the movements to be performed and then physically executing these same movements after. Action Observation Therapy has already been shown to be appropriate for the telehealth setting [11].

The pilot feasibility study researching the use of Action Observation Therapy in persons with mid-portion AT, was implemented remotely for 12-weeks, making it a telehealth study. Telehealth refers to the delivery of healthcare over distance via information and communication technology [12]. The use of telehealth has been in practice since the 1990s, however exponential growth has been seen in this sector due to the limiting impact of the COVID-19 pandemic on traditional in-person consultations. Systematic reviews have indicated the positive impact telehealth has had on physical function, disability, pain and satisfaction levels in people with musculoskeletal conditions [13]. To date, only two studies has explored the management of Achilles Tendinopathy via telehealth and overall positive satisfaction and acceptability levels were found [3, 14].

The Medical Research Council (MRC) guidance for developing complex interventions acknowledge qualitative research is of particular value during the feasibility stage of a trial development and can identify important learning for the full trial [2, 15]. Qualitative research gives us access to patients’ perspectives, beliefs, experiences and behaviour [16], which can hugely inform future research and clinical practice [17]. Welcoming and acknowledging our patients viewpoints is equally important for patient-centred care [18] and for establishing the acceptability of a trial intervention. As properties of a health intervention along with the interactions between an intervention and its context can contribute to complexities [15], this complexity is further reinforced when there is no previous research to guide the implementation of the intervention.

This study has two aims; (1) to explore the participants in the intervention groups experience of using Action Observation Therapy and to further ascertain the acceptability of this intervention (2) to establish the participants in the control and intervention groups experience of participating in a telehealth study, the motivators for participation and opinions of the trial processes.

## Methods

### Study design

This was a qualitative follow-up study undertaken on a group of participants from a previous pilot feasibility randomised controlled trial which explored the effect of Action Observation Therapy with Eccentric loading in the treatment of mid-portion AT over the course of 12- weeks [19]. Two groups of participants with mid-portion Achilles Tendinopathy were compared; the intervention group watched videos of the eccentric exercises to be performed prior to performing the same exercises, while the control group watched videos of landscapes before performing the two same eccentric exercises.

### Recruitment

All participants (*n* = 24) who completed the 12-week pilot feasibility study were invited to participate in the qualitative study. This purposive selection was necessary to allow access to the participants experiences and perspectives of the trial. Follow-up emails were sent to the participants who initially consented to partake in the qualitative study at the time of completion of the pilot feasibility trial, and all participants who responded to the email were enrolled.

### Data collection

The interviews were conducted within three months of the final participant completing the pilot feasibility study. Participants were informed of the reasons for conducting the research. Semi-structured interviews were conducted online using topic guides (Appendix 1). The topic guide explored both the trial processes and the experience of the Action Observation Therapy intervention. Following the interview participants were invited to add any information that they felt was of importance and had not been discussed. All interviews were conducted by the chief investigator in the pilot feasibility trial, an experienced musculoskeletal Physiotherapist who had completed the Oxford qualitative research methods course and is a current PhD student (DR). Interviews were transcribed verbatim through the videoconference software (Zoom Video Communications) [20].

### Data analysis

All transcripts were cross-checked with the audio recordings and corrected accordingly. A reflexive thematic analysis as guided by Braun and Clarke [21] was used to identify patterns of meaning in relation to the research aims, allowing for inductive theme generation and both descriptive and interpretative portrayals of the data. Importantly this form of analysis recognises and embraces the positionality of the researcher, recognising their own story and acknowledges the influence that this has on perceiving and sculpting the data [21]. The six stages outlined in this analysis approach include; (i) data familiarisation through immersion in the data whereby the transcripts were read multiple times (ii) coding via the systematic engagement with the data providing both rigour and insight. To provide a richer level of understanding, three transcripts were coded by another member of the research team (CO’S) and understandings and meanings were discussed (iii) generating initial themes (iv) developing and reviewing and naming themes (v) candidate themes and subthemes were presented and discussed with a consensus reached amongst other members of the research team (CO’S, ER) (vi) The final step was the write-up. Nvivo 12 software (QSR International Limited) was used in the coding phase providing structure and organisation of the data. The consolidated criteria for reporting qualitative research (COREQ) was used to guide the reporting of this study.

## Results

### Participants

Sixteen participants (ten female and six male) were recruited; nine in the intervention group and seven in the control group. Ages ranged from 28- 58 years (mean age 45), the mean BMI was 26, and the mean duration of symptoms was 12-months (Table 1). Varying physical activity levels from recreational walkers to iron men competitors were seen, ranging from high to low on the International Physical Activity Questionnaire. Duration of interviews ranged from 11—41 min with a mean duration of 19 min.

**Table 1:** Participant characteristics

Participant number	Group allocation	Gender	Age	BMI	IPAQ	Duration of symptoms (months)	Prior history	Worst NPRS at time of entry to pilot study
2	Intervention	Female	41	29.2	Moderate	10	No	4
4	Intervention	Male	28	22.59	Moderate	24	No	4
7	Intervention	Female	56	25.85	Moderate	18	No	6
8	Intervention	Male	43	21.03	Moderate	3.5	Yes	4
10	Intervention	Female	52	40.56	Moderate	7.5	No	9
11	Intervention	Female	44	20.78	Moderate	> 24	No	5
23	Intervention	Female	35	20.76	Moderate	3	No	3
24	Intervention	Male	48	24.49	High	7	No	8
29	Intervention	Female	44	27.48	High	14	No	8
13	Control	Male	45	24.98	High	3.5	No	3
15	Control	Female	44	29.75	Low	11	No	4
17	Control	Male	37	25.22	High	3	Yes	8
20	Control	Female	50	22.31	Moderate	4	No	5
22	Control	Female	43	26.93	Moderate	3.5	No	5
26	Control	Female	45	20.38	High	18	No	8
30	Control	Male	58	32.87	High	36	No	7

*BMI* Body Mass Index, *IPAQ* International Physical Activity Questionnaire, *NPRS* Numerical Pain Rating Scale

## Results and discussion

A total for five themes and one subtheme were identified from the data; (i) *The impact of Achilles Tendinopathy is commonly not prioritised* with the sub-theme *the acceptance and minimisation of pain*; (ii)*Therapeutic alliance has the greatest impact on support;* (iii) *Factors which influenced adherence;* (iv) *Action Observation Therapy is valued and recommended and* (v) *Recommendations for future interventions.*

### The impact of Achilles Tendinopathy is commonly not prioritised

Theme one relates to the second aim, to understand the motivators for trial participation. The primary motivators for study participation were ‘good timing of the study’ and ‘an opportunity to try something to help’, which created the impression that a sense of chance led to the treatment. In other words, had the study not occurred the participants would not have otherwise actively sought out treatment or created the opportunity themselves to have the injury treated.

*‘it just kind of was like oh gosh you know, maybe this is a good time to just actually treat this and see what the story is or see if it is an issue because it’s just been there niggling away’(Participant (P) 11).*

*‘It was just after the Achilles had gotten really bad and I saw a post somewhere on social media, and I thought why not give it a go and see if it makes a longer-term difference. It was just the timing really’(P4).*

*‘so yeah I think I said look it I'll try something different, and so that was that was the reasoning.’ (P8)*

A trend was seen where the Achilles tendon had been previously treated but non-specifically or not as the priority in the treatment sessions. That the reason for attending the session was for another injury and the Achilles Tendinopathy ‘*not the sole purpose’* of attending the session.

*‘But it was the first time where I would have treated that specifically, it would have been more just, so I was going for a general rub-down and I’d have said to say the physical therapist oh my Achilles is bothering me there’ (P11).*

*‘I don’t know if I ever specifically did go for that I think as a direct treatment this was the first time’ (P26).*

Similarly, a non-committal approach to historic treatments is seen whereby those who had actively attended treatment for the Achilles injury did so in a non-committal fashion as the rehab was ‘*let go by the wayside’* or only went to physiotherapy once or twice. It has already been reported in the literature that a significant number of patients, either never engage in or otherwise never complete treatment courses for chronic pain [22]. In Ireland alone, it has been found that 25% of people with chronic muscle, joint or osteoarthritic pain did not seek help and a proportion of those who did waited up to two years before doing so [23]. Similarly, a large proportion of adults in the UK living with chronic musculoskeletal pain did not access health services for their knee pain [24, 25].

*‘I suppose first time treating it as a recurring treatment. Might have gone for physio once or twice before and then it just cooled down after.’ (P4).*

*‘just in and out of a couple of people just giving it rubs and whatever’ (P7)*

Despite a prior review citing that women are more likely than men to seek treatment for chronic pain [26], no difference was noted in this study with both genders displaying a similarly casual approach to seeking help. Whilst a lack of motivation has been described as a reason why persons with Achilles Tendinopathy do not seek help [6], motivation was not identified here as all participants were sufficiently motivated to join and complete the 12-week study. The barrier seemed to be a self- belief that the injury wasn’t bad enough to warrant treatment or that they could continue to put up with it as they had done so this far. This approach to health seeking or delay in seeking rehabilitation may be contributing to the chronicity of this condition and associated poor treatment outcomes.

#### Sub-theme; acceptance and minimisation of pain

This sub-theme is a continuation of the aim to establish motivators to participation in the trial. Despite the average pain Numerical Pain Rating Score at the time of enrolment into the study being 5/10, only two participants stated pain was the motivating factor in joining the study. Mixed reports surround the impact of pain on help-seeking behaviours whereby severity of pain has been shown not to be associated with help seeking behaviours [27], while another study found pain severity to have an influence on help seeking behaviours but not as much mobility difficulties [28]. This study has similar findings as impairments were more commonly referenced as the motivating reason to join.

There appeared to be an acceptance of a moderate level of pain amongst the participants. While the acceptance of pain can be considered a positive, the belief that the pain cannot be altered or changed is not a beneficial way to conceptualise pain [29]. The acceptance of this pain amongst participants is predominantly captured as one putting up with the pain and accepting this is how it is despite the limiting impact the pain was having.

*‘some days the pain might be ten some days it might be five and I got to the stage where I kind of thought right I’m just going to have to live with this’ (P10).*

*‘because it wasn’t really impacting hugely on my life other than not being able to run or whatever, I don’t know whether I would have gone and looked for- I probably just would have let it niggle away and hoped that it got better itself’(P23).*

Another participant insightfully shares that they would have waited for it to get worse before going to physiotherapy; ‘*I’d probably be in a lot of pain if I hadn’t sorted it out you know, and I probably would have had to go to Physio, it would have taken longer you know because I would have waited till that point, you know to get it’.(P15).*

*‘you know by ignoring it or by just kind of learning to live with us because it wasn't so bad that I couldn't function so that sort of you know you're in that in between sort of place where you're living with something quite well so, but then it's back your head you're going ummm you know should I be doing something about this.’ (P11).*

The language used to describe pain gives insight into the participants perspective of the pain and the story being told about the pain experience [30], the kind of relationship one has with their pain and how they experience their suffering [31]. The word niggle is commonly used the describe the pain. Our belief is such a description not only minimises the impact of the presence of pain but also trivialises the pain experience. Some examples of this are seen above and further exerts are provided below:

*‘the mental side of kind of having that sort of niggle and just that fear in the back of your head that you're doing more damage all the time’ (P11)*

*‘when I ran, the niggle I was always mindful I was always trying to mind it if you know what I mean’ (P13)*

*‘I think I was probably a little bit embarrassed to go to the doctor, because I was like oh it's only a niggle it's only an niggle and it was during lockdown and everybody was like you can’t go to the doctor unless you’re you know about to check out I guess.’ (P20).*

This endurance of pain represents a stoicism and has been previously reported in participants with posterior tibial tendon dysfunction who similarly ‘just got on with it’ despite the pain [32]. Similarly, the minimisation of pain has also been reported in the literature with reference to knee pain as individuals accepted it as normal part of ageing [27].

Recognising that many sufferers of Achilles Tendinopathy are not likely to actively seek treatment emphasises the need for healthcare professionals to proactively target this cohort with the goal of normalising access to healthcare for all levels of pain. Educating Achilles Tendinopathy sufferers that any functional impairment is worthy of treatment, and the fact that one has tolerated the limitations and levels of pain severity so far, isn’t sufficient a reason to keep doing so.

### Therapeutic alliance greatly impacts support

This theme relates to the second aim, to understand the participants’ experience of participating in a telehealth study. Therapeutic alliance refers to the working relationship between the participant and the clinician [33], which in this case is the clinical researcher (DR). The quality of this relationship is associated with therapeutic success [34]. The level of support provided, professionalism, communication, education and personalisation presented as important factors for participants in forming a strong therapeutic alliance, each will be explored below. No single participant felt less supported in the study despite there being no face-to-face contact at any stage. This echoes the findings of Badawy et al. [35] who found the feasibility, accessibility, satisfaction and health outcomes of telehealth services to be comparable with or better than in-person services. The predominant reason provided for feeling supported was due to the support given by the clinical researcher.

*‘I felt honest to God I could pick up the phone or email you at the drop of a hat if I ever needed to.’*

*‘I did not feel at any time kind of isolated, I felt the support was always there.’ (P2).*

Knowing that someone was there and not feeling alone seemed to be pivotal in providing a sense of support and reassurance; ‘ *you told me if I had any questions to contact you and that you’d get back to me straightaway, so I knew I wasn’t left on my own that I knew I had a point of contact at any stage.’ (P20).* This resulted in all participants feeling the level of support would have been the same had the study been conducted in-person.

*‘I definitely didn’t feel like I wasn’t supported anyway and I definitely don’t think the online aspect lessened it in any factor or lessened it in any way you know’ (P17).*

Professionalism and confidence in the level of the skill presented as further qualities which further facilitated the positive therapeutic alliance. This confidence and trust in the treating therapist have also reported in a systematic review as factors which influenced patient-therapist interactions in musculoskeletal physical therapy [36].

*‘ A professional kind of element of support and reassurance, and it was really positive and I think that really helped with my commitment and my rehabilitation, you know the journey so I felt like it was a better experience because of your input’. (P15).*

*‘having someone with your medical know how and your professionalism and experience was a godsend to me’ (P20).*

Communication was described as another factor which seemed to strengthen the therapeutic alliance and positively contributed to participants feeling supported. An important aspect in communication was clarity and participants felt this allowed a good understanding of the respective roles and collaboration between. This is consisted with the findings from O’Keefe et al. [36] whom reported patients felt their interaction with the therapist was enhanced when they knew what the treatment plan was.

*‘It was very clear from the outset what was involved, what your role was going to be, what my role was going to be’ (P10).*

*‘it was very clear, there was no elements of confusion from my side, so I knew what was expected of me’ (P17)*

*‘it was very thorough in terms of how you felt before, exactly what you were going to do and you know assessing you afterward’ (P26)*

Participants also appreciated the educational aspect of the communication, finding the information interesting. Regular information and education has previously been shown to have a positive impact of patients’ perception of the quality of treatment received [37]. Effective communication is recognised as an essential skill that clinicians should master to improve upon the quality of care [38].

*‘the information was very helpful I did actually find the science very interesting to know what was happening’ (P2).*

*‘I’m big into how things work and that, so I would always be interested anytime I go to a Physio as the mechanism as to how things work so yeah I was very interested in it’ (P8)*

*‘you know it was reassuring to kind of have that um I suppose the backing of the science behind it and kind of the knowledge that you’re in safe hands that was important to me’ (P15).*

The value in improving participants education and knowledge through informative communication is seen in this study as no participant was threatened by the pain experienced during the exercises as they were educated about this pain and knew it was a normal and welcomed response to the exercises. This is very interesting considering kinesiophobia, pain catastrophising thoughts and central sensitisation were identified in some of the participants at baseline. Littlewood et al., [39] similarly reported that patients were not concerned by the pain experience during exercise rehabilitation for Achilles Tendinopathy. Patient education is intended to influence the patients ability to cope with impairment through knowledge [40] and the delivery of education appeared to positively influence the participants subsequent experience of the pain. Maladaptive beliefs and avoidance behaviours have previously been reported with persons with Achilles Tendinopathy [6] and this study illustrates the power of education with targeting maladaptive beliefs in persons with this condition. The importance of learning to deal with pain and how to build tolerance for physical activity has been previously expressed by participants with low back pain [41]. This is further supported by a systemic review and meta-analysis which found pain education strategies to be effective in reducing both pain intensity and disability in chronic musculoskeletal pain [42].

*‘doing them again in the evening there might be a bit of pain and you know I knew you had said that’s normal and you know to keep doing them’ (P11).*

*‘it was sore you know and obviously you did say to be look it will feel a bit sore while you’re doing them but then I was kinda going yeah it’s got a bit easier’ (P13)*

Participants appreciated feeling the care received was personalised. The importance of a personal aspect to care for the overall quality of the therapeutic relationship has previously been reported by both physiotherapists and patients with musculoskeletal complaints [43]. The authors found this enabled a recognition of the participants as people with lives outside of the clinical study whilst also respecting professional boundaries. This importance of feeling personally valued was also described as an important aspect for participants participating in neurologic rehabilitation [44], while the systematic review described how patients who felt they were treated as just another person and not individually recognised reported a negative impact on the interaction they had with their therapist [36].

*‘ I found it very very personal and like I developed a rapport with you straight away’ (P10).*

*‘I found interacting with you absolutely fantastic and like just the personalisation of it was really good because you know when I was asking you questions you were directly addressing issues for me’ (P17).*

Therapeutic alliance positively influence treatment results including treatment adherence [45], reductions in pain and disability and leads to higher satisfaction levels in musculoskeletal Physiotherapy [46]. Understanding what participants perceive to be important contributors to a positive therapeutic alliance offers immense value as this will allow therapists to actively address, cultivate and build upon these skills to ensure a good-working relationship where patients with Achilles Tendinopathy feel supported and valued. Appreciating that patients feel sufficiently supported doing physiotherapy sessions remotely opens up opportunities for both the clinician and the patient whereby time, geographical or transport limitations would have prevented receiving physiotherapy treatment and guidance in the past.

### Factors which influenced adherence

This theme relates to both aim one and two, it provides insight into both participants’ opinions of the trial processes and the acceptability of the intervention. Adherence is described as the extent to which a person’s behaviour corresponds with the recommendations provided by a the healthcare professional [47]. Factors which influence the degree to which participant and patients follow rehabilitation programmes can be considered as barriers or facilitators [48]. Starting and then continuing adherence to a programme require behavioural change [49] and three main factors were found to positively influence adherence in this study; the accountability provided by the application (app), the check-ins with the therapist and seeking or maintaining the benefit from the rehabilitation.

Salaso was the app used in the pilot feasibility study which allowed participants to log-in and access their rehabilitation programmes. Fifteen of the participants liked using the app with, ‘easy’, ‘straight-forward’ and ‘ really good’ being the common descriptors used. The final participant described themselves as a paper person and for that reason preferred using a diary over the app. Salaso can be accessed by logging into the web-page or downloading the application, and 14 of the participants downloaded the app onto their phone. Participants felt the app was helpful and encouraged them to continue doing the rehabilitation programme with the structure provided appealing to participants. Changizi & Kaveh [50] describe how the benefits of technology in rehabilitation are not confined to the objective monitoring of the individuals’ adherence, but can extend to stimulate ones engagement in the rehabilitation, as was evident in this study.

*‘It’s just having the app is good, it gives you something you need to tick off every day so you tend to not skip days’ (P4)*

*‘the fact that you’re loading them onto an app I think that’s great because it kinda puts a little bit of pressure on you to make sure you get it done (P13).*

*‘that just suited me because I felt right box ticked you know, I like to tick things off a plan or a programme, you know I like that feeling when I’ve ticked it.’(P11).*

*‘Like it was probably one of the reasons I did it was just so I could log it’ (P17)*

Participants with musculoskeletal conditions who received their home exercise program on an app with remote support have been previously shown to have greater adherence and also function when compared to those participants who just received paper hand-outs [51]. Participants felt having the app encouraged exercise fidelity. The app prevented retrospectively logging exercise after midnight for a given day. This prevented any form of back-reporting or hoarding which has been found is ePaper diaries [52].

*‘there was no kind of hiding or you know there’s no kind of I can just pretend I’ve done these’ (P11)*

*‘I like the way you couldn’t cheat if you didn’t enter in real time so if kept you honest’ (P24).*

Participants valued the check-ins with the clinical researcher and felt these were a source of encouragement to continue in the study. Previously, the regularity of contact has also been described as important factor for maintaining a commitment to exercise in adherence levels in telehealth for patients with chronic pulmonary disease [53] and Achilles Tendinopathy [3].

*‘I think if we hadn’t had those check-ins, if we just had the one at the start and the one at the end probably my compliance and commitment wouldn’t have been as much as it was’(P2).*

*‘if we didn’t have the six week check-in I don’t know if I have done as well at the end to be honest, because that you know kind of revisiting everything, that’s a confidence booster and gives you that extra push’(P10).*

*‘I remember doing the six week check-in with you, where the function had improved a lot and definitely that would have just given me great confidence that this was working and to keep on going’(P11).*

Seeking the perceived benefit of the rehabilitation was described as a motivating stimulus for participants to continue with the trial. Similarly, participants also found the positive results achieved as a result of the rehabilitation programme motivated them to continue adhering to the programme. The resulting positive outcomes can convince one of the need to sustain the improved activity [54]. The positive response to therapy was also shown to be a main factor influencing engagement in rehabilitation to rotator cuff tendinopathy [39].

*‘I didn’t want to do it in a way that was half-hearted because I wouldn’t get the benefit and then I’d wonder well maybe it’s because I didn’t do it right’ (P 15)*

*‘ for me it was a case of this is definitely worth continuing to try and resolve this once and for all’ (P30)*

*‘I could see the improvements, I could see that it was working, that I could do more heel raises, and so I think that kind of kept me motivated to keep on going’ (P23)*

*‘you could see that you were moving up the weights but you weren’t getting fatigued so was a real case of you’re staying on top of this, also you know you’re getting stronger’(P24)*

Up to 70% of patients do not perform home exercise plans as prescribed [55]. Motivation has been shown to influence both physical activity [56] and participation in training [57] and so recognising sources for motivation is valuable in encouraging adherence in patients. Understanding that the use of an app is a welcomed and positive addition for participants, that regular check-ins with the therapist further contribute to adherence and seeking or maintaining the positive changes from rehabilitation programmes encourage patients to continue to commit to their rehabilitation programmes offers great insight. Perhaps summarising and re-enforcing the positive changes as and when they occur in rehabilitation would be beneficial to the patient for ongoing adherence.

### Action Observation Therapy is valued and recommended

This theme relates to first aim, exploring participants’ experience of using Action Observation Therapy and ascertains the acceptability of this intervention. All participants in the Action Observation Therapy group were satisfied with the treatment they received, and most participants in this group felt their expectations were met or exceeded.

*‘The whole thing exceed my expectations and I’ve got a spring in my step, I have an exercise program that I have continued since it finished that helps me and that has improved my quality of life and I would highly recommend this to anyone.’ (P10).*

*‘yeah definitely, yeah surpassed I would say really, I sort of thought it was there so long you know that it’s kind of just a fact a life.’ (P11)*

*‘ I would have said way exceeded you know, as I said before I’ve gone back to playing at a level I played at in my 20 s’ (P24)*

Two of the participants who felt their desires to return to pre-injury running weren’t met, both felt there may have been other contributing factors.

*‘I would say partially met because right not today I haven’t gone back running since we were last talking, I have done small bits of running but I haven’t gone back to the level I was at. But I think that’s also because I’ve changed how I was exercising over summer, like I did a huge amount of hiking and the ankle never held me back.’ (P2).*

*‘but it mightn’t be just because of the Achilles it might be the other things going on.’(P4)*

The main changes described were decreased or complete resolution of pain on activity decreased, strength and mobility improvements and decreases in stiffness.

*‘yeah yeah there’s no pain it’s great.’ (P23).*

*‘I got more function mobility, I suppose I noticed it more on the steps. You know I tended to walk the steps on the tippy toes and I noticed it there that I start to walk one side equal to the other.’ (P4).*

*‘The reduction on pain on what I would call normal things, as I’ve said earlier on just been able to walk around the house doing housework without getting sore or going up and down stairs without hobbling.’ (P7).*

Participants also describe the positive impact on mental well-being experienced due to participating in the study. The negative impact of Achilles Tendinopathy on psychological well-being has been previously reported, with a loss of self-identity described as a result of the activity limitations [5, 6]. Similarly, Slagers et al. [58] reported an association with psychological factors and tendon severity, function and participation in persons with Achilles and patellar tendinopathy. It is known that living with chronic pain can result in or worsen symptoms of anxiety and depression [59].

*‘this chronic Achilles it’s been going on for two years or however long, two to three years so yeah I felt you mentally I suppose this gave a lift, I was doing something proactive.’*

*‘you’re in a much better place at week 6 and not just physically, but as I said to you before like mentally you felt better because you are going out and getting your exercises done.’ (P24).*

Being able to return to wearing certain footwear was an another area where participants described meaningful changes. This captured a meaningful outcome measure for some participants which wasn’t formally assessed in the quantitative study. Physiotherapists have been active in developing and supporting the use of outcome measures [60]. Psychometric properties of an outcome measure such as the reliability and validity are important, and choosing measures that possess adequate properties is considered a primary concern for practitioners [61]. However, what changes are meaningful for our patients should also be captured, this ensures our care is always patient-centred, there is little value is adhering to globally adopted outcome measure sets if they do not carry the patients goals too. Understanding the whole patient and their goals allows care plans to address the patient’s needs [62].

*‘I was able to buy a nice pain of sandals this summer and wear them without knowing I will be in absolute agony for a couple of days afterwards because I’ve kind of spend the last two or three years in trainers all the time and yeah you can buy nice comfortable trainers but there’s nothing nicer than wearing a nice pain of sandals and having your nails pained and you know standing tall and feeling good about yourself.’ (P10).*

*‘I’m back to wearing like runners and shoes that I gave up wearing that used to aggravate my foot because I used to find the weight of the runner was that little bit too heavy, I’m back to wearing all the footwear that I used to wear before, well bar one pair of boots, before I ever had an issue with it like which is amazing.’ (P29).*

All participants in the intervention group found the videos a helpful addition to their rehabilitation programme. This is an important discovery as the participants were not informed of the mirror neuron system for the duration of the study yet found watching the videos a beneficial component of their rehabilitation plan.

*‘I always think you need the videos to do it properly because I find that when somebody is explaining it to you, what they are saying and what the reality is aren’t necessarily the same thing.’ (P2).*

*‘it makes you aware that you’re doing the technique correctly, do you know what I mean. Even if you think you’ve done one set not quite right, that second set you are watching more what you’re doing.’ (P7).*

*‘But I always just found that watching them at the start always made sure that my body my legs were in the right position, the way they should be. Because again if you do something wrong, you could injure yourself more but no, I was a big fan of watching videos I always did them.’ (P29).*

*‘I felt the videos were perfect there was no bells or whistles with it, it was exactly this is what you have to do, this is how you do it, and it was very well demonstrated so, for me it was perfect.’ (P10).*

As per the MRC framework, both the feasibility and acceptability of interventions can be improved by the engagement of any potential intervention user [15]. Specifically to this study, the acceptability for using Action Observation Therapy in persons with Achilles Tendinopathy was established by engaging and interviewing the participants. All participants in the intervention group recommended this rehabilitation, establishing high levels of acceptability for this intervention, this could expand treatment possibilities for future Achilles Tendinopathy sufferers.

*‘II would highly recommend this to anybody’ (P10)*

*‘would recommend it to anybody who has ongoing Achilles issues.’ (P11)*

*‘Oh my God 100%, I mean if you’re trying to avoid an operation it’s the only thing to probably do.’ (P29)*

### Considerations for future studies

This final theme relates to the first and second aim, as it built upon the participants’ experiences of using Action Observation Therapy, the telehealth aspect and the trial processes. Patient and participant feedback relates to the recording of the patients’ perspective on the quality of care received in order to improve both the processes and the patients experience [63]. Additionally, the nature of pilot feasibility studies is the evaluation of trial design and processes to inform larger randomised controlled trials [64]. Therefore participants feedback from this study will greatly inform any future subsequent research. Suggestions provided for improvements related to the app, the assessments and the videos.

Regarding the Salaso app three participants felt it would have been beneficial to be able to log the exercises past midnight, which they found limiting.

*‘I might be going to bed that night and it had just gone past midnight and I’d go sugar I hadn’t put in the thing but it was now gone you know’ (P2).*

*‘yeah that was the one drawback that you couldn’t retrospectively log a day’ (P8).*

One further suggestion in relation to the app was to have the function of being able to view your history.

*‘I would have liked to have been able to see the history to show what I had logged and what I hadn’t logged’ (P2)*

*‘it doesn’t actually tell you in the last hundred days you’ve completed the exercise X number of times.’ (P24)*

Another participant felt it might be a motivating feature if the app informed you in a positive way how many sets you had completed. Both the quantity and quality of feed-back delivered to patients has been shown to influence motivation levels in patients [65].

*‘You've done one of your video watches already, you've only got two left to do today.*

*Do You know, like if it had phrased it that way, it might be better’ (P15)*

Suggestions provided in relation to the assessments included having an in-person session at the beginning of the study, having more assessments scheduled throughout the study and reducing the number of questionnaires in the assessment. The most common suggestion was to have an in-person session at the beginning of the study, participants felt it would have provided further value by either doing the assessment in person or going through how to navigate the app and do the exercises in person. It is important to highlight that an initial in-person session was in the original design of the study but had to be changed due to the COVID-19 pandemic.

*‘the face to face maybe that would be beneficial as part of the assessment at the start’ (P11).*

*‘maybe initially you have a talk face-to-face where you go through and take over their screen and kind of say now you click on this and then you do the exercises, show me the exercise, Okay and now you click on this’. (P15).*

*‘possibly one meeting for the description of what to do, I suppose like a normal Physio appointment would have been more ideal’. (P26)*

Participants also felt having at least one more meeting, so the second assessment is earlier would be beneficial. This offer great insight as researchers are cognisant of the time demands placed upon participants in clinical trials and so it is beneficial to know that participants found these assessments offered value and utility and that participates would have happily given more time to have more assessments.

*‘I don’t know would it have been more benefit to have more, certainly not less. Maybe four, eight and 12-weeks rather than just the six and 12’. (P11).*

*‘ It was really useful to have those connects and I would say maybe you could have even one or two more’ (P15)*

*‘yeah I think it was a good gap I think between them in the middle, like you know and you know it’s sometimes harder to keep going with the exercises with maybe a longer gap, a shorter gap maybe or something in between might be better’. (P22).*

Reducing the number of questionnaires in the assessment was suggested, although participants were understanding of the importance of data collection in research. The selection of questionnaires implemented was guided by the International Scientific Tendinopathy Symposium Consensus (ICON) group [66], with the Widespread Pain Index and symptom severity questionnaire and a satisfaction questionnaire added.

*‘like there were a good few different surveys like, so I think maybe a little bit less you know’. (P15).*

*‘if I was trying to improve maybe a process like that maybe lessen the amount of questions’. (P17).*

Whilst a further participant was happy with the number of questionnaires they suggested keeping notes to have a record of their previous answers would have been helpful to have in the subsequent assessments.

‘the only issue I would have is that you’d early want to take notes from one assessment to the next to know what your assessment points were because there was certain times when say on the second assessment that I wouldn’t have remembered what score I gave myself the first time’. (P8).

In relation to the video content used for the Action Observation Therapy intervention,four participants felt as time went on continuing to watch the videos was tedious and the repetition was challenging. As previously mentioned, participants were not informed of the mirror neuron system and it is not possible to say whether knowledge of why the videos were being watched would have altered this sense of tedium.

*‘I think it was just the cumulative amount of time that was the challenging thing you know’ (P2).*

*‘say I watched it fully at the start and then that tailed off because it was the same thing’ (P4).*

*‘it could be a bit tedious do you know what I mean, sometimes it was hard to make myself just focus on watching them between each set’. (P7)*

Solutions provided by participants to combat this were, only watch the video once at the beginning of each set or to just watch them in the first portion of the rehabilitation programme. Separately, another participant who found the repetition of watching the videos tedious, couldn’t think of an aspect that could be changed to improve this.

*‘ I think I would have gotten more value from watching the first one first and then when I was doing the second one watching that then and not watching them in between…. I can understand why there might be good reason why you do it just at the start of each session’ (P2).*

*‘they were very helpful at the start, certainly for making sure you got your technique right and they were good to remind you of the technique but as I said once towards the end and you were so used to doing it, the technique had become so ingrained that you didn’t need them as much I thought.’ (P8).*

Some participants starting watching the videos and doing the exercises concurrently and this could potentially offer the solution to the tedium and time challenges. The simultaneous performance of physical exercises whilst watching videos has previously prescribed in other Action Observation Therapy studies [67–69]. The research team were cognisant of the volume of video watching prescribed and felt while there was the risk of over-prescription as no previous data existed in relation to action observation therapy and Action Observation Therapy, a reasonable first step was to watch the video prior to each set.

*‘I used to just put the phone on the third or fourth step and sort of do at the same time as watching the videos’ (P11).*

*‘For the first while, I put them on before I did it, but then like a week into it or further than that I’d put it on while I was doing it, and it would kind of remind me of little kind of things that you might slip up on’ (P23).*

Patient and public involvement in research involves inclusion in shaping the design of research, participants recommendations is a powerful contributor to this process [70]. The suggestions provided by the participants are critical for eventual translational research [71] and will accordingly guide the re-evaluation process of both the trial processes and Action Observation Therapy intervention.

## Considerations and limitations

It is important to highlight the positionality and subjectivity of the researcher in reflexive thematic analysis. Each researcher has their own story, life experiences and view-points and it is through this individual lens that the participants interviews have been perceived and understand [72]. The generative role that the researcher plays in thematic analysis is recognized and welcomed and it is equally appreciated that the data would be interpreted differently and tell a different story to another research team.

Additionally, the timing of the pandemic meant that many participants had already adjusted to the world of online meetings and appointments, this likely positively impacted conducting the study online. Perhaps had participants not had this acclimatisation period to teleconferences prior to joining the study, the sentiment may have been different and future studies conducted remotely may not be so graciously received by participants.

The interviews were conducted after the trial was completed and therefore relied on the retrospective memory of the participants, which may have introduced a recall bias. While the participants included in this study are representative of the typical patient characteristics seen in mid-portion Achilles Tendinopathy in terms of age, BMI and duration of symptoms [3, 4, 6], only two-thirds of the participants who completed the study were interviewed, therefore the experiences and perspectives of the unincluded participants may differ and contrast to the narratives captured in this study. In accordance with previous qualitative research which explored participants experience of participating in the trial, there was a reluctance of participants to criticise the rehabilitation experience [32, 44]. Having the trial investigator conduct the interviews may also have introduced a source of reporting bias. Despite encouragement for all feed-back be it positive or negative, there was the tendency to minimise any negative aspects. This in part may have been due to the fact that the same researcher from the 12-week study conducted the interviews and participants may have been reluctant to complain.

## Conclusion

This study contributes to the pool of qualitative research in the field of Achilles Tendinopathy and is the first to explore participants experiences of Action Observation Therapy and only the second to explore telehealth as a delivery method in this condition. Considerations for futures studies based upon the findings of this study include legitimising all levels of pain and to encourage individuals with Achilles Tendinopathy to action treatment. Healthcare professionals treating Achilles Tendinopathy should be aware of the important impact which therapeutic alliance has on the degree of support provided to patients, and this can be maintained and cultivated remotely and treatments need not be in-person in the clinic. Adherence to rehabilitation is positively influenced by using an app, having regular check-in sessions with the Physiotherapist and reinforcing the positive changes in outcome measures can assist this. Recognising that Action Observation Therapy is valued and recommended informs us that Action Observation Therapy is considered a beneficial and acceptable treatment by patients with Achilles Tendinopathy. This opens up possibilities for future Action Observation Therapy treatment for persons with Achilles Tendinopathy and the suggestive feed-back provided by the participants will allow the treatment provided to be further improved.

## Supplementary Information

**Additional file 1: Appendix 1.** Topic Guide.

## Data Availability

All consent forms and transcripts will be held in a locked cabinet for 3 years as per the criteria specified in the ethics form.

## References

[1] van der Plas A, de Jonge S, de Vos RJ, van der Heide HJ, Verhaar JA, Tol JL (2012). A 5-year follow-up study of Alfredson’s heel-drop exercise programme in chronic mid-portion Achilles tendinopathy. Br J Sports Med.

[2] O’Cathain A, Hoddinott P, Lewin S (2015). Maximising the impact of qualitative research in feasibility studies for randomised controlled trials: guidance for researchers. Pilot Feasibility Stud.

[3] Hasani F, Malliaras P, Haines T (2021). Telehealth sounds a bit challenging, but it has potential: participant and physiotherapist experiences of gym-based exercise intervention for Achilles tendinopathy monitored via telehealth. BMC Musculoskelet Disord.

[4] Mallows A, Head J, Goom T, Malliaras P, O'Neill S, Smith B (2021). Patient perspectives on participation in exercise-based rehabilitation for Achilles tendinopathy: A qualitative study. Musculoskelet Sci Pract.

[5] McAuliffe S, Synott A, Casey H, Mc Creesh K, Purtill H, O'Sullivan K (2017). Beyond the tendon: Experiences and perceptions of people with persistent Achilles tendinopathy. Musculoskelet Sci Pract.

[6] Turner J, Malliaras P, Goulis J, Mc Auliffe S (2020). "It's disappointing and it's pretty frustrating, because it feels like it's something that will never go away." A qualitative study exploring individuals' beliefs and experiences of Achilles tendinopathy. PLoS One.

[7] Rizzolatti G, Craighero L (2004). The mirror-neuron system. Annu Rev Neurosci.

[8] Peng TH, Zhu JD, Chen CC, Tai RY, Lee CY, Hsieh YW (2019). Action observation therapy for improving arm function, walking ability, and daily activity performance after stroke: a systematic review and meta-analysis. Clin Rehabil.

[9] Ryan D, Fullen B, Rio E, Segurado R, Stokes D, O'Sullivan C (2021). Effect of Action Observation Therapy in the Rehabilitation of Neurologic and Musculoskeletal Conditions: A Systematic Review. Arch Rehabil Res Clin Transl.

[10] Sarasso E, Gemma M, Agosta F (2015). Action observation training to improve motor function recovery: a systematic review. Arch Physiother.

[11] Molinaro A, Micheletti S, Pagani F, Garofalo G, Galli J, Rossi A, Fazzi E, Buccino G (2022). Action Observation Treatment in a tele-rehabilitation setting: a pilot study in children with cerebral palsy. Disabil Rehabil.

[12] Darkins A, Cary M, Association Free, Books; London, (2000). Telemedicine and Telehealth: Principles.

[13] Cottrell MA, Galea OA, O’Leary SP, Hill AJ, Russell TG (2017). Real-time telerehabilitation for the treatment of musculoskeletal conditions is effective and comparable to standard practice: a systematic review and meta-analysis. Clin Rehabil.

[14] Post A, Eboinie RK, Sluka KA, Moseley L, Bayman E, Hall M (2022). Effectivenss of telehealth for achilles tendinopathy on pain, function, and pain-related psychological outcomes during COVID-19. J Pain.

[15] Skivington K, Matthews L, Simpson SA, Craig P, Baird J, Blazeby JM, Boyd KA, Craig N, French DP, McIntosh E, Petticrew M, Rycroft-Malone J, White M, Moore L (2021). A new framework for developing and evaluating complex interventions: update of Medical Research Council guidance. BMJ.

[16] Slade SC, Patel S, Underwood M, Keating JL (2018). Rigorous qualitative research in sports, exercise and musculoskeletal medicine journals is important and relevant. Br J Sports Med.

[17] Braun V, Clarke V (2019). Novel insights into patients' life-worlds: the value of qualitative research. Lancet Psychiatry.

[18] Kvåle K, Bondevik M (2008). What is important for patient centred care? A qualitative study about the perceptions of patients with cancer. Scand J Caring Sci.

[19] Ryan D, O’Donoghue G, Rio E (2022). The effect of combined Action Observation Therapy with eccentric exercises in the treatment of mid-portion Achilles-tendinopathy: a feasibility pilot randomised controlled trial. BMC Sports Sci Med Rehabil.

[20] Video Conferencing, Web Conferencing, Webinars, Screen Sharing - Zoom. Available: https://zoom.us/.

[21] Braun V, Clarke V (2022). Thematic Analysis A practical guide.

[22] Turk DC, Rudy TE (1991). Neglected topics in the treatment of chronic pain patients—relapse, noncompliance, and adherence enhancement. Pain.

[23] Veale DJ, Woolf AD, Carr AJ (2008). Chronic musculoskeletal pain and arthritis: impact, attitudes and perceptions. Ir Med J.

[24] Bedson J, Mottram S, Thomas E, Peat G (2007). Knee pain and osteoarthritis in the general population: what influences patients to consult?. Fam Pract.

[25] Moore AJ, Gooberman-Hill R (2020). Why don't patients seek help for chronic post-surgical pain after knee replacement? A qualitative investigation. Health Expect.

[26] Cornally N, McCarthy G (2011). Help-seeking behaviour for the treatment of chronic pain. Br J Community Nurs.

[27] Jinks C, Ong BN, Richardson J (2007). A mixed methods study to investigate needs assessment for knee pain and disability. Population and individual perspectives. BMC Musculoskelet Disord..

[28] Thorstensson CA, Gooberman-Hill R, Adamson J, Williams S, Dieppe P (2009). Help-seeking behaviour among people living with chronic hip or knee pain in the community. BMC Musculoskelet Disord.

[29] McCracken L, Vowles K (2006). Acceptance of chronic pain. Curr Pain Headache Rep.

[30] Munday I, Kneebone I, Newton-John T (2021). The language of chronic pain. Disabil Rehabil.

[31] Bourke J (2012). Languages of pain. Lancet.

[32] Campbell RF, Morriss-Roberts C, Durrant B, Cahill S (2019). "I need somebody who knows about feet" a qualitative study investigating the lived experiences of conservative treatment for patients with posterior tibial tendon dysfunction. J Foot Ankle Res.

[33] Peplau HE (1997). Peplau's theory of interpersonal relations. Nurs Sci Q.

[34] Taylor RR, Lee SW, Kielhofner G, Ketkar M (2009). Therapeutic use of self: a nationwide survey of practitioners' attitudes and experiences. Am J Occup Ther.

[35] Badawy SM, Barrera L, Sinno MG, Kaviany S, O'Dwyer LC, Kuhns LM (2017). Text messaging and mobile phone apps as interventions to improve adherence in adolescents with chronic health conditions: a systematic review.. JMIR Mhealth Uhealth.

[36] O'Keefe M, Cullinane P, Hurley J, Leahy I, Bunzli S, O'Sullivan PB, O'Sullivan K (2016). What influences patient-therapist interactions in musculoskeletal physical therapy? Qualitative systematic review and meta-synthesis. Phys Ther.

[37] Del Baño-Aledo ME, Medina-Mirapeix F, Escolar-Reina P, Montilla-Herrador J, Collins SM (2014). Relevant patient perceptions and experiences for evaluating quality of interaction with physiotherapists during outpatient rehabilitation: a qualitative study. Physiotherapy.

[38] Mauksch LB, Dugdale DC, Dodson S, Epstein R (2008). Relationship, communication, and efficiency in the medical encounter: creating a clinical model from a literature review. Arch of Intern Med.

[39] Littlewood C, Malliaras P, Mawson S, May S, Walters S (2014). Patients with rotator cuff tendinopathy can successfully self-manage, but with certain caveats: a qualitative study. Physiotherapy.

[40] van Koulil S, van Lankveld W, Kraaimaat FW, van Helmond T, Vedder A, van Hoorn H, Donders R, de Jong AJ, Haverman JF, Korff KJ (2010). Tailored cognitive-behavioural therapy and exercise training for high-risk patients with fibromyalgia. Arthritis Care Res.

[41] Unsgaard-Tøndel M, Søderstrøm S (2021). Therapeutic Alliance: Patients' Expectations Before and Experiences After Physical Therapy for Low Back Pain-A Qualitative Study With 6-Month Follow-Up.. Phys Ther.

[42] Marris D, Theophanous K, Cabezon P, Dunlap Z, Donaldson M (2021). The impact of combining pain education strategies with physical therapy interventions for patients with chronic pain: a systematic review and meta-analysis of randomized controlled trials. Physiother Theory Pract.

[43] Miciak M, Mayan M, Brown C, Joyce AS, Gross DP (2018). The necessary conditions of engagement for the therapeutic relationship in physiotherapy: an interpretive description study. Arch Physiother.

[44] Wain HR, Kneebone II, Billings J (2008). Patient experience of neurologic rehabilitation: a qualitative investigation. Arch Phys Med Rehabil.

[45] Hall AM, Ferreira PH, Maher CG, Latimer J, Ferreira ML (2010). The influence of the therapist-patient relationship on treatment outcome in physical rehabilitation: a systematic review. Phys Ther.

[46] Fuentes J, Armijio-Olivo S, Funabashi M, Miciak M, Dick B, Warren S, Rashiq S, Magee DJ, Gross DP (2014). Enhanced therapeutic alliance modulates pain intensity and muscle pain sensitivity in patients with chronic low back pain: an experimental controlled study. Phys Ther.

[47] De Geest S, Sabat E (2003). Adherence to long-term therapies: evidence for action. Eur J Cardiovasc Nurs.

[48] Bassett SF (2020). Bridging the intention-behaviour gap with behaviour change strategies for physiotherapy rehabilitation non-adherence. N Z J Physiother.

[49] Teo JL, Zheng Z, Bird SR (2022). Identifying the factors affecting 'patient engagement' in exercise rehabilitation. BMC Sports Sci Med Rehabil..

[50] Changizi M, Kaveh MH (2017). Effectiveness of the mHealth technology in improvement of healthy behaviors in an elderly population—a systematic review. mHealth..

[51] Lambert TE, Harvey LA, Avdalis C, Chen LW, Jeyalingam S, Pratt CA, Tatum HJ, Bowden JL, Lucas BR (2017). An app with remote support achieves better adherence to home exercise programs than paper handouts in people with musculoskeletal conditions: a randomised trial. J Physiother.

[52] Stone AA, Shiffman S, Schwartz JE, Broderick JE, Hufford MR (2002). Patient non-compliance with paper diaries. BMJ.

[53] Hoaas, H, Andreassen H.K, Lien L.A (2016). Adherence and factors affecting satisfaction in long-term telerehabilitation for patients with chronic obstructive pulmonary disease: a mixed methods study.. BMC Med Inform Decis Mak.

[54] Breckenridge JP, Gray N, Toma M (2019). Motivating Change: a grounded theory of how to achieve large-scale, sustained change, co-created with improvement organisations across the UK. BMJ Open Quality.

[55] Beinart NA, Goodchild CE, Weinman JA, Ayis S, Godfrey EL (2013). Individual and intervention-related factors associated with adherence to home exercise in chronic low back pain: a systematic review. Spine J.

[56] Morris JH, Oliver T, Kroll T, Joice S, Williams B (2017). Physical activity participation in community dwelling stroke survivors: synergy and dissonance between motivation and capability A qualitative study. Physiotherapy.

[57] Signal N, McPherson K, Lewis G, Kayes N, Saywell N, Mudge S (2016). What influences acceptability and engagement with a high intensity exercise programme for people with stroke? A qualitative descriptive study. Neurorehabilitation.

[58] Slagers AJ, van Veen E, Zwerver J, Geertzen JHB, Reininga IHF, van den Akker-Scheek I (2021). Psychological factors during rehabilitation of patients with Achilles or patellar tendinopathy: a cross-sectional study. Phys Ther Sport.

[59] Tüzün EH (2007). Quality of life in chronic musculoskeletal pain. Best Pract Res Clin Rheumatol.

[60] Kane R (1994). Looking for physical therapy outcomes. Phys Ther.

[61] Beattie P (2001). Measurement of health outcomes in the clinical setting: applications to physiotherapy. Physiother Theory Pract.

[62] Kitson A, Marhsall A, Bassett K, Zeitz K (2013). What are the core elements of patient-centred care? A narrative review and synthesis of the literature from health policy, medicine and nursing. J Advanced Nurs..

[63] Golda N, Beeson S, Kohli N (2018). Analysis of the patient experience measure. J Am Acad Dermatol.

[64] Blatch-Jones AJ, Pek W, Kirkpatrick E (2018). Role of feasibility and pilot studies in randomised controlled trials: a cross-sectional study. BMJ Open.

[65] Colombo R, Pisano F, Mazzone A (2007). Design strategies to improve patient motivation during robot-aided rehabilitation. J Neuroeng Rehabil.

[66] Vicenzino B, de Vos R, Alfredson H (2020). ICON 2019—International Scientific Tendinopathy Symposium Consensus: There are nine core health-related domains for tendinopathy (CORE DOMAINS): Delphi study of healthcare professionals and patients. Br J Sports Med.

[67] Cowles T, Clark A, Mares K (2013). Observation-to-Imitate Plus practice could add little to physical therapy benefits within 31 days of stroke: translation randomized controlled trial. Neurorehabil Neural.

[68] Hsieh Yw, Lin YH, Wu CY, Lin YP, Chen CC. Treatment Effects of Upper Limb Action Observation Therapy and Mirror Therapy on Rehabilitation Outcomes after Subacute Stroke: A Pilot Study. Behav Neurol. 2020:6250524. 10.1155/2020/6250524 PMC7199557 32377266

[69] Tung M, Murphy I, Griffin S (2014). Observation of limb movements reduces phantom limb pain in bilateral amputees. Ann Clin Transl Neurol.

[70] Dudley L, Gamble C, Allam A, Bell P, Buck D, Goodare H (2015). A little more conversation please? Qualitative study of researchers’ and patients’ interview accounts of training for patient and public involvement in clinical trials. Trials.

[71] Sacristán JA, Aguarón A, Avendaño-Solá C, Garrido P, Carrión J, Gutiérrez A, Kroes R, Flores A (2016). Patient involvement in clinical research: why, when, and how. Patient Prefer Adherence.

[72] Denzin NK, Lincoln YS, Denzin  NK, Lincoln YS (2005). Introduction: The discipline and practice of qualitative research. The SAGE handbook of qualitative research (2^nd^ ed., oo.1–32).

