# Supplementary material for: “I’ve got a spring in my step” participants experience of action observation therapy and eccentric exercises, a telehealth study for mid-portion Achilles Tendinopathy: a qualitative study
Source: J Foot Ankle Res. 2023 Apr 11;16:19. doi: 10.1186/s13047-023-00619-x (PMC10088142; doi:10.1186/s13047-023-00619-x)
Supplement: Supplementary file 1 — Additional file 1: Appendix 1. Topic Guide. [file 13047_2023_619_MOESM1_ESM.docx]

| **All**  **Appendix 1:** Topic Guide | **Background** | 1. Was this your first time treating your Achilles tendinopathy? |
| --- | --- | --- |
| **All** | **Recruitment** | 1. What encouraged you to participate in this study/what attracted you to the study? 2. How did you find the recruitment process? |
| **All** | **Assessment & Support** | 1. The study involved being assessed online at three different time points using physical tests and a range of questionnaires. How did you find the assessment process? 2. The assessments were conducted online which meant there was no face-face interaction at any stage in the study, how did you find this? |
| **All** | **Rehabilitation Process** | 1. Please talk me through your process of rehabilitation/   Tell me about your experience of the rehabilitation? |
| **Intervention Group** | **Videos** | 1. I understand, watching the videos was a part of your intervention, tell me about your experience of watching the videos? 2. In relation to the videos, do you feel there is anything which could be improved upon? 3. Do you feel you watched the videos as often as prescribed? |
| **All** | **Exercises** | 1. How did you find doing the strengthening exercises? 2. Do you feel you did the exercises as frequently as prescribed/ how often did you do the exercises? |
| **All** | **Salaso App** | 1. How did you find using the Salaso application?   (Prompt anything you particularly liked or disliked)   1. Did you download Salaso as an app or continue to log into the webpage? 2. Salaso has the feature of logging when you had completed your exercises, did you find you used this feature? |
| **All** | **Expectations & Satisfaction** | 1. What were your expectations of the weeks of rehabilitation. 2. Did you feel your expectations were met? 3. How would you describe your level of satisfaction with the treatment you received?   (Prompt…What was the main reason for this?) |
|  | **Results** | 1. What changes, if any did you notice after doing the 12 weeks rehabilitation?   (Prompt.. Discuss what the outcome measures assessed) |
|  | **Closing Questions** | 1. Would you recommend this form of rehabilitation? 2. Is there anything further which you feel we should know or that you would like to add? |
